# ADDRESSING THE THEORETICAL GAPS IN ELDER FAMILY FINANCIAL EXPLOITATION

**DOI:** 10.1093/geroni/igz038.1409

**Published:** 2019-11-08

**Authors:** Megan Gilligan, Axton Betz-Hamilton, Ashton Chapman

**Affiliations:** 1 Iowa State, Ames, Iowa, United States; 2 South Dakota State, Brookings, South Dakota, United States; 3 Robert Wood Johnson Foundation, Princeton, New Jersey, United States

## Abstract

A systematic review of elder family financial exploitation (EFFE) literature from the past five years reflects limited use or consensus of theoretical perspectives. In this paper, we propose using Bronfenbrenner’s Bioecological Theory to frame the dynamic, interrelated factors associated with EFFE. Bronfenbrenner’s Bioecological Theory, specifically the PPCT model includes Proximal processes, Person characteristics, Contextual systems, and Time. Proximal processes are increasingly complex interactions between individuals (e.g., family communication). Person characteristics include demand (e.g., gender, age), resource (e.g., education), and force (e.g., temperament) characteristics affecting interactions. Contextual levels drawn from Bronfenbrenner’s original model (e.g., micro, meso, exo, and macro) emphasize the effect of interrelated systems on development. T refers to Time, including changes occurring in time (e.g., longitudinal) and over time (e.g., historical). Collectively, the PPCT model provides a framework for understanding the iterative, complex factors linked to EFFE.

